# Tinnitus Treatment with Oxytocin: A Pilot Study

**DOI:** 10.3389/fneur.2017.00494

**Published:** 2017-09-21

**Authors:** Andreia Aparecida Azevedo, Ricardo Rodrigues Figueiredo, Ana Belen Elgoyhen, Berthold Langguth, Norma De Oliveira Penido, Winfried Schlee

**Affiliations:** ^1^Universidade Federal de São Paulo, São Paulo, Brazil; ^2^Faculdade de Medicina de Valença, Valença, Brazil; ^3^Instituto de Investigaciones en Ingeniería Genética y Biología Molecular “Dr. Héctor N. Torres”, CONICET, Buenos Aires, Argentina; ^4^Facultad de Medicina, Instituto de Farmacología, Universidad de Buenos Aires (UBA), Buenos Aires, Argentina; ^5^Department of Psychiatry and Psychotherapy, University of Regensburg, Regensburg, Germany

**Keywords:** tinnitus, oxytocin, hearing disorders, pharmacotherapy, nasal sprays

## Abstract

**Introduction:**

Tinnitus is the perception of sound in the absence of an external stimulus. It is a frequent condition for which there is as yet no pharmacological treatment approved. Auditory and non-auditory pathways are involved in tinnitus’ pathophysiology. Oxytocin is a neurohormone and eventual neurotransmitter that plays a complex role in social cognition and behavior.

**Objective:**

To evaluate the potential of oxytocin as a tinnitus treatment.

**Study design:**

Two studies were performed. Study 1 was a long-term open pilot study, while study 2 investigated short-term effects with a double-blinded placebo-controlled cross-over study.

**Setting:**

Ambulatory ENT care.

**Subjects and method:**

In study 1, 15 patients were investigated over a 10-week period in an open pilot study. In study 2, 16 patients were included in a placebo-controlled crossover trial to investigate short-term effects following a single dose.

**Results:**

For the long-term study (study 1), analysis of variance revealed a significant decrease in tinnitus sensation, both for the Tinnitus Handicap Inventory and Clinical Global Impression (CGI). Also, the short-term effects in study 2 revealed a significant reduction of tinnitus because of the oxytocin nasal spray as measured with the Visual Analog Scale and the CGI Scale.

**Conclusion:**

These preliminary studies demonstrated that oxytocin may represent a helpful tool for treating tinnitus and further larger controlled studies are warranted.

## Introduction

Tinnitus is a phantom auditory sensation that is not generated by an external stimulus. It is a symptom that affects about 25% of American adults, in a frequent basis for around 8% of them (1). According to current trends of thoughts, tinnitus is a central nervous system phenomenon that follows an initial peripheral damage and may be modulated by many concurring neuronal circuits (2). Neuroimaging studies suggest that tinnitus results from the dynamic interaction between auditory and non-auditory pathways, and the result of this interaction, especially when the limbic and autonomous systems are involved, is the trigger of negative emotional associations and appearance of uncomfortable reactions (3). As yet, there is no Food and Drug Administration (FDA)-approved pharmacological treatment for tinnitus. However, various factors structures such as ion channels, neurotransmitters, and receptors have been proposed to be involved in tinnitus pathophysiology (4). Thus, there is no reasonable argument to believe that tinnitus could not be pharmacologically treated (5).

Oxytocin is a neurohormone that may also act as a neurotransmitter, produced by magnocellular neurons in the ventricular nuclei of the hypothalamus (6). Oxytocin’s production is stimulated by high estrogen doses and inhibited by high doses of catecholamines (dopamine, epinephrine, and norepinephrine) (7). It is released to the bloodstream by the posterior pituitary. Oxytocin receptors (OTR) are widespread throughout the human body and belong to the family of heterotrimeric G-protein-coupled receptors, which are found in many cell types (8). OTR promote the inhibition of adenilate-cyclase, thus reducing intracellular levels of cyclic-AMP and subsequently opening potassium channels and closing calcium channels, having, thereby, an inhibitory signature (9).

Oxytocin is responsible for important physiological functions, such as uterine contraction, lactation stimulation, sperm transport, and ejaculation (10). In addition, oxytocin plays a complex role in social cognition and behavior. Important aspects of human social interaction such as empathy, trust, and social learning are influenced by oxytocin (11). Among other mechanisms the pro-social effects of oxytocin are mediated by reduction of amygdale activation (12). Recently, it has been shown, that oxytocin also increases the salience of acoustic social stimuli by modulating the inhibitory function in the auditory cortex (13).

Imaging studies have demonstrated that tinnitus loudness and tinnitus distress are reflected by increased activation in networks involving the auditory cortex and the amygdala, respectively (3, 14). Therefore, we hypothesized that oxytocin may influence tinnitus perception and tested in two studies whether its intranasal application reduces tinnitus loudness and distress.

## Materials and Methods

The trial comprised 2 studies, as follows.

### Study 1

#### Study Design

Study 1 was designed as an open pilot study.

#### Subjects

Fifteen patients who presented with the primary complaint of tinnitus in a local ENT clinic in São Paulo and Valença were included. Only patients older than 18 years with a continuous perception of tinnitus of at least 6 months duration were included. Patients were excluded if their Tinnitus Handicap Inventory (THI) (15) score in the Brazilian Portuguese validated version (16) was below 16 points. All patients underwent a complete otolaryngological evaluation and were excluded from the study in case of external or media otitis or in the presence of A-s, B, and C tympanogram curves. Also, women in fertile age [up to 49 years old (17)] were excluded from the study, to avoid oxytocin-induced uterine contractions. Informed consent was obtained prior to the study, and the patients were informed about the clinical procedures. Clinical and demographic characteristics of participants (mean age, percentage of females, mean duration of tinnitus in months, laterality and scores on the THI in both studies) are described in Table 1.

**Table 1:** Patient groups, clinical and demographic data (mean ± SD).

*n*	Age	Female (%)	Duration of tinnitus in months	Laterality	THI at baseline
Study 1 (*n* = 15)	60.6 ± 9.6	40	107.8 ± 118.7	5 unilateral	56.8 ± 28.2
				7 bilateral	
				3 in head	
Study 2 (*n* = 16)	62.8 ± 10.6	31.3	78.8 ± 147.9	11 unilateral	52.8 ± 30.5
				5 bilateral	

*THI, Tinnitus Handicap Inventory*.

#### Experimental Procedure

Oxytocin (Syntocinon) was administered daily for a duration of 10 weeks. Patients were instructed to apply one puff [each puff corresponds to 4 oxytocin IU] of oxytocin in each nostril two times a day, which sums up to a dosage of 16 IU per day. The patients were asked to store the oxytocin in the refrigerator throughout the study period. Subsequent visits were made in weeks 1, 2, 3, 4, 6, 8, and 10 to monitor study progress and screen for side effects. Treatment effects were assessed with the THI (primary outcome measurement) and the Clinical Global Impression (CGI) scale improvement [secondary outcome measurement; CGI-I (18)]. The CGI scale is a well-accepted instrument in clinical research for evaluating the clinical improvement retrospectively. The instruction in the CGI is: “Please rate the total improvement of your tinnitus complaints compared to before beginning of treatment.”

#### Statistical Analysis

For the THI, a mixed model analysis of variance (ANOVA) was calculated with the factor time and a random intercept per patient. A mixed model ANOVA was also calculated for the CGI with the factor time (weeks 1–10) and a random intercept per patient. In case of positive results of the ANOVA further comparisons were calculated.

### Study 2

During study 1, five subjects reported an immediate effect (5–10 min after drug administration) on tinnitus sensation (around 50% reduction of tinnitus volume). Considering this unexpected finding, we decided to investigate this tinnitus reduction following one single dose in more detail. Therefore, we performed a second study with a single dose of oxytocin treatment in a double-blind placebo-controlled study using a crossover design. As study 1 turned out with positive results, study 2 was performed with a similar number of patients.

#### Subjects

Study 2 was performed in the same ENT clinic as study 1. Seventeen patients with the primary complaint of tinnitus and who had not participated in study 1 were included.

As in the first study, all patients were older than 18 years and reported chronic tinnitus of more than 6 months duration. Prior to the study, all patients were seen by an experienced ENT doctor. Patients with vascular or muscular origin of their tinnitus, patients with conductive hearing loss and patients with regular intake of other medications during the study period were not included. Patients reporting previous experience with oxytocin nasal spray were not included as well. A further inclusion criterion was a tinnitus loudness rating of at least 4, measured on a scale between 0 and 10.

One patient dropped out of the study because she could not return for the second visit. Clinical and demographic characteristics of the 16 patients, which were included in the analysis, are displayed in Table 1. Informed consent was obtained prior to study participation.

#### Experimental Procedure

In study 2, the effects of nasal oxytocin administration were compared with placebo treatment. The order of drug treatment was counterbalanced. Patients were randomized to receive either a single dosage administration of 16 IU of oxytocin (Syntocinon) or a placebo treatment with a nasal spray containing distilled water, which is indistinguishable to oxytocin nasal spray considering smell and taste. A randomization table was created prior to study start by Winfried Schlee who is not in contact with the study participants. After an interval of 1 week, the patients received a second administration with either placebo (the group who received oxytocin first) or with oxytocin (the group who received placebo first). The patients, as well as the medical doctor who evaluated the results (author 1), were blinded to the treatment assignment. Author 2, who applied oxytocin and placebo, was not blinded. In the oxytocin condition, patients received two puffs of oxytocin in each nostril, which corresponded to 16 IU. Likewise, in the placebo treatment, the patients received two puffs distilled water in each nostril, which is supposed to have no clinical effect.

The primary outcome measurement was a visual analog scale (VAS) asking for the tinnitus loudness (19). The VAS score for tinnitus loudness was assessed directly before administering the nasal spray as well as 30 min and 24 h after the intervention. Additionally, the CGI-I was applied 30 min and 24 h after the intervention as secondary outcome measurement.

#### Statistical Analysis

For the VAS scores a two-way mixed model ANOVA was calculated with the factors *time* and *treatment*. A random intercept was modeled for each participant. Mixed model ANOVA were also used to analyze the results of the CGI scale. For the CGI scores, again a two-way mixed model ANOVA was calculated with the factors *time* and *treatment*.

The statistical analysis was performed with the statistical software package R, version 3.3.3 [R Core Team (2017). R: a language and environment for statistical computing. R Foundation for Statistical Computing, Vienna, Austria. URL https://www.R-project.org/]. For the mixed models analysis, the “nlme” library (version 3.1-131) was used. A *p*-value ≤ 0.05 was considered as significant. Correction for multiple comparisons was applied using the stepwise Holm–Bonferroni method (20).

#### Study Registration

The study was approved by the ethics committee and registered at http://clinicaltrials.gov.

## Results

### Study 1

**Figure 1:**
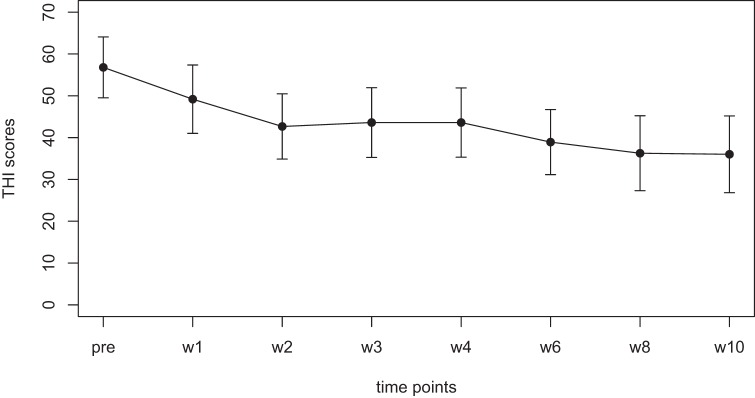
Average scores of the Tinnitus Handicap Inventory (THI) (0–100) before the treatment and at all visits during the study (weeks 1–8). The final assessment was done at the end of treatment in week 10.

**Figure 2:**
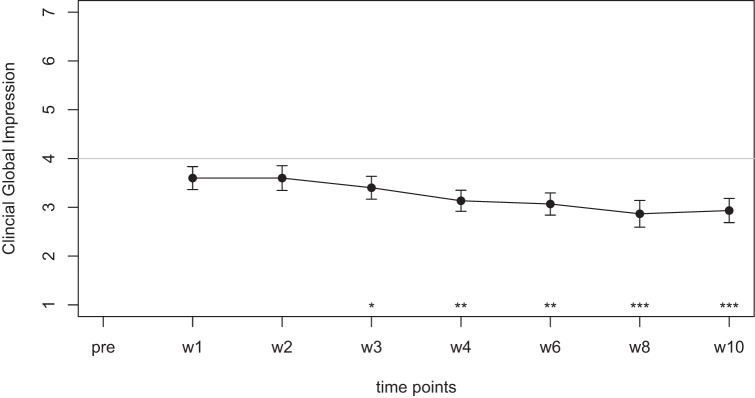
Average scores of the Clinical Global Impression scale (1–7). A rating of 4 on this scale indicates “no change.” Smaller numbers indicate an improvement of clinical symptoms (1 = “very much better,” 2 = “much better,” 3 = “minimally better”).

Mean scores for the THI and the CGI-I are presented in Figures 1 and 2, respectively. Both measures show continuous and significant decreases of tinnitus-related symptoms measured with the THI [*F*(7,98) = 7.45, *p* < 0.0001] as well as global clinical improvement (CGI-I) [*F*(6,84) = 5.22, *p* < 0.0001] over the study period.

For the THI scores, further comparisons using paired *t*-tests between baseline and all other time points reveal significant differences for the THI measurements at week 1, 2, 3, 4, 6, 8, and 10 (*p*-values of all comparisons survived the Holm–Bonferroni method for multiple comparisons).

For the CGI-I additional tests were calculated to test if scores were different from the value of 4, which indicates “no change.” No significant difference was found for week 1, week 2, and week 3. The comparisons for week 4, 6, 8, and 10; however, survived the Holm–Bonferroni correction for multiple comparisons. For week 4 and 6, the CGI scores indicated a change with high significance (*p* < 0.01), for week 8 and 10, the CGI scores indicated a change with very high significance (*p* < 0.001).

Paired *t*-tests were calculated to compare the maximum hearing loss of the participants before and after the treatment. The hearing function did not change significantly, neither for the right ear (*p* = 0.2) nor for the left ear (*p* = 0.36).

### Study 2

**Figure 3:**
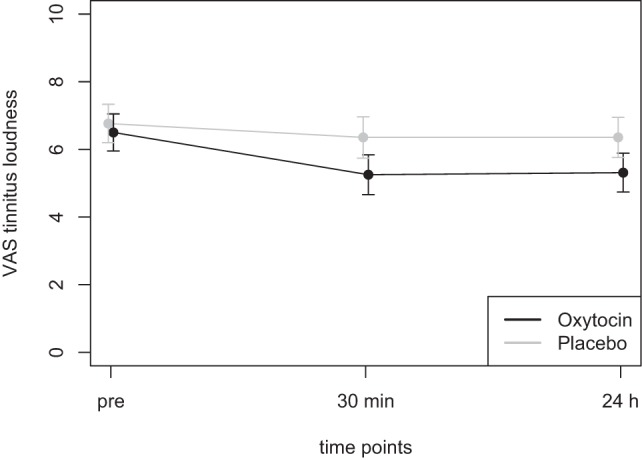
Average scores of the Visual Analogue Scale (0–10) before the treatment and at two time points after the intervention (30 min and 24 h after).

**Figure 4:**
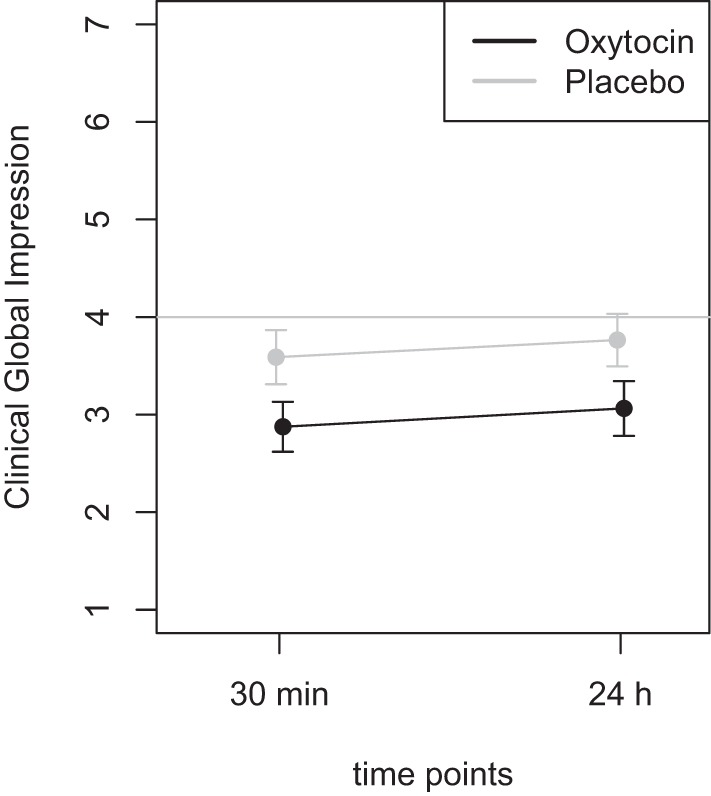
Average scores of the Clinical Global Impression scale (1–7). A rating of 4 on this scale indicates “no change.” Smaller numbers indicate an improvement of clinical symptoms (1 = “very much better,” 2 = “much better,” 3 = “minimally better”).

Mean scores of the oxytocin treatment and the placebo intervention are presented in Figures 3 and 4 for the VAS scores and the CGI-I, respectively.

In the ANOVA for the VAS scores, the main effect of *treatment* was highly significant [*F*(1,77) = 9.54, *p* = 0.003] and the main effect of *time* was significant [*F*(2,77) = 4.47, *p* = 0.015]. The interaction effect *time* × *treatment*, however, failed to reach the level of significance [*F*(2,77) = 1.13, *p* = 0.33]. Cohen’s *d* effect sizes for the VAS ratings were 0.55 (30 min) and 0.56 (24 h) for oxytocin treatment and 0.18 (30 min) and 0.19 (24 h) for placebo treatment, respectively.

Furthermore, the ANOVA using the CGI scores as dependent variable, the main effect *treatment* was highly significant [*F*(1,46) = 8.72, *p* = 0.005], while the main effect *time* did not reach statistical significance [*F*(1,46) = 0.60, *p* = 0.44]. There was no statistically significant interaction effect [*F*(1,46) = 0.001, *p* > 0.9]. Additional *t*-tests were calculated to confirm the results and showed that there was no statistical significant difference between the two time points (30 m and 24 h after treatment) for the oxytocin (*p* > 0.63) nor for the placebo condition (*p* > 0.58). A *t*-test testing for the difference between the oxytocin and placebo intervention, irrespective of the time point, revealed a statistically significant improvement for the oxytocin treatment (*t* = −2.9, *p* = 0.005).

## Discussion

The present results show that in an exploratory non-controlled study, a 10-week treatment with daily intranasal puffs of oxytocin produced a significant reduction in the THI and CGI in a group of tinnitus patients. Moreover, a double-blind controlled study with a single dose of an intranasal oxytocin, although not significant, exhibited a tendency in a reduction in tinnitus distress, as measured by the CGI.

The presented pilot studies were designed to explore possible effects of oxytocin on tinnitus. The open 10 week treatment study (study 1) suggests that regular intake of oxytocin can reduce tinnitus-related handicap. Open pilot studies have been suggested as a useful screening tool to identify potentially promising pharmacological compounds (21–23). Motivated by the promising results of study 1, we performed also a placebo-controlled crossover trial to investigate the short-term effects of a single dose of oxytocin. Whereas questionnaires represent the gold standard for assessing longer lasting effects of therapeutic interventions (24), short-term effects can be best detected with VAS. While the clinical effects of the short-term oxytocin treatment are much smaller than the long-term effects in study 1, they still suggest a potential therapeutic role of oxytocin for tinnitus. These preliminary results warrant further controlled studies, which should include more patients and investigate chronic treatments with oxytocin. The results of the presented pilot studies provide an estimation of the effect sizes for both acute and long-term effects of oxytocin to inform the study design of future randomized controlled studies. While the open study with the long-term treatment (study 1) revealed a strong reduction of tinnitus-related distress over time, the short-term treatment (study 2) revealed only small differences between placebo and oxytocin. Further studies on oxytocin treatment for tinnitus should invest in long-term treatment.

A wide variety of compounds are used off-label to treat tinnitus patients (5, 25). However, there is still no US FDA- or European Medicines Agency-approved drug on the market for this clinical unmet use (5). The comprehensive list of compounds includes almost the entire pharmacopeia arsenal, such as anxiolytics, anticonvulsants, antidepressants, *N*-methyl d-aspartate (NMDA) antagonists, cholinergic antagonists, antihistamines, vasodilators, antipsychotics, sodium and calcium channel antagonists, antidiuretics, and herbal medicines, among others (5, 25). In most cases, the pharmacological treatment is used to treat comorbidities which accompany tinnitus, such as frustration, annoyance, anxiety, depression, irritation, concentration difficulties, and sleep disturbances which are most relevant for the perceived tinnitus severity (5, 19). In this context, the exploratory use of oxytocin on tinnitus patients is well justified, since it is a compound with a different mechanism of action from those previously tested.

In particular, Kirsch et al. have shown that when compared with placebo, oxytocin potently reduces activation of the amygdala and coupling of the amygdala to brainstem regions implicated in autonomic and behavioral manifestations of fear (26). In this regard, tinnitus neuroimaging techniques have identified brain networks related to tinnitus associated distress which include the amygdala and the autonomic nervous system (3). Recently, it has been shown that tinnitus distress correlates with enhanced effective connectivity from the amygdala to the auditory cortex (27). An EEG analysis revealed that tinnitus distress is correlated with more synchronized alpha activity in various emotion-related areas, including the subcallosal anterior cingulate cortex, the insula, the parahippocampal area and the amygdale (28). In addition, an MEG functional connectivity analysis has shown long-range coupling between frontal, parietal and cingulate brain areas in alpha and gamma phase synchronization related to tinnitus distress (29). In summary, tinnitus distress has been related to the co-activation of a network which includes amygdala, anterior cingulate cortex, insula, parahippocampal area and which is under direct influence of the posterior cingulate and prefrontal cortex (30, 31). This network partially overlaps with brain areas implicated in distress in patients suffering from pain (32), dyspnea in asthma (33), somatoform disorders (34) and might, therefore, represent a non-specific distress network.

Through source localized resting-state EEG and electrocardiogram recordings, the participation of the autonomic nervous system to tinnitus distress has been investigated, showing that the dorsal and subgenual anterior cingulate, as well as the left and right insula are important in the central control of the autonomic system in tinnitus patients (35, 36). The perceived distress in tinnitus patients seems to be sympathetically mediated. It is of interest that the same areas that are involved in the control of the autonomic system are also involved in salience processing and distress. This suggests that the autonomic nervous system critically influences salience perception and distress associated with tinnitus.

Thus, the observed reduction of tinnitus handicap after long-term oxytocin application could be explained by a modulatory influence of oxytocin on amygdala activity and on a decoupling between amygdala activation and autonomous nervous system regulation. Further studies investigating the effect of oxytocin on perception and simultaneously on brain activity and connectivity could shed further light on the mechanisms by which oxytocin interacts with tinnitus perception and distress.

## Ethics Statement

Comitê de Ética em Pesquisa Médica do Centro Universitário de Volta Redonda—Unifoa/Fundação Parecer No. 42257214.1.0000.5237/1.017.546 7/4/2015.

## Author Contributions

Study design: AA, RF, WS, BL, and AE. Data collection: AA and RF. Statistics: WS. Writing: AA, RF, AE, BL, and NP. Manuscript revision: AA, RF, AE, BL, and PS. All contributed for writing and revision: AA, RF, BL, AE, NP, and WS.

## Conflict of Interest Statement

The authors declare that the research was conducted in the absence of any commercial or financial relationships that could be construed as a potential conflict of interest.
